# Assistência de Inteligência Artificial na Análise de Artigos Médicos por Pares: Vantagens, Desvantagens e Considerações Éticas

**DOI:** 10.36660/abc.20250315

**Published:** 2025-08-12

**Authors:** André Zimerman, Andre d’Avila

**Affiliations:** 1 MOVE Academic Research Organization Hospital Moinhos de Vento Porto Alegre RS Brasil MOVE Academic Research Organization, Hospital Moinhos de Vento, Porto Alegre, RS- Brasil; 2 Hospital SOS Cardio Florianópolis SC Brasil Hospital SOS Cardio, Florianópolis, SC – Brasil

**Keywords:** Revisão por Pares, Inteligência Artificial, Ética, Publicações

Recentemente, os autores deste artigo “Ponto de Vista“ foram convidados a revisar por pares um manuscrito para um prestigiado periódico cardiovascular internacional. Após dedicar alguns dias à análise, eles decidiram, por curiosidade, inserir o manuscrito em um modelo de linguagem de inteligência artificial (IA) comercialmente disponível com o seguinte prompt: “Analise e Critique o Arquivo Anexo”. Ambos os revisores ficaram surpresos com a resposta gerada em segundos pelo algoritmo de IA — sua profundidade e precisão superaram suas expectativas, com alguns insights se mostrando complementares aos seus. Como resultado, eles incorporaram esses elementos à revisão oficial submetida ao periódico.

Essa experiência, no entanto, levantou outra questão: eles deveriam reconhecer a assistência fornecida pelo algoritmo de IA? Sem saber a resposta, eles consultaram outros médicos que revisam regularmente manuscritos para periódicos de alto impacto, e suas opiniões variaram bastante. Alguns argumentaram que a IA é apenas uma ferramenta para aumentar a eficiência, não exigindo reconhecimento — os revisores são responsáveis pelo conteúdo final e devem usar quaisquer estratégias que lhes permitam alcançar o melhor resultado. Outros insistiram que não divulgar a assistência da IA seria antiético.

Para resolver o dilema, os autores recorreram novamente à própria IA para obter insights sobre seu papel no processo de revisão por pares de manuscritos médicos. A resposta, apresentada aqui, exigiu ajustes editoriais mínimos por parte dos autores. *Sempre que necessário, as contribuições dos autores são relatadas ao longo do manuscrito em itálico* .

## IA fala sobre o papel da IA como revisora de artigos médicos:

O processo de revisão por pares é um pilar fundamental da integridade científica, particularmente na área da medicina, onde uma avaliação rigorosa garante que a pesquisa publicada seja confiável, reproduzível e segura para aplicação clínica. No entanto, o crescente volume de artigos médicos submetidos para publicação, aliado à complexidade da pesquisa moderna, tem sobrecarregado o sistema tradicional de revisão por pares.

O uso de IA na revisão por pares de artigos médicos representa uma oportunidade transformadora para enfrentar os crescentes desafios da publicação acadêmica em uma área onde precisão, pontualidade e confiabilidade são primordiais. No entanto, além de seus potenciais benefícios, a IA apresenta desvantagens significativas e desafios notáveis. Este ensaio examina os benefícios e as deficiências da IA na análise por pares de artigos médicos, bem como as implicações éticas de sua integração.

### Vantagens da IA na revisão por pares

1. **Eficiência e velocidade aprimoradas** O volume de pesquisas médicas submetidas para publicação aumentou acentuadamente, muitas vezes sobrecarregando revisores humanos e atrasando a disseminação de descobertas cruciais. A IA pode processar grandes quantidades de texto rapidamente, realizando tarefas como verificação de referências, análise de dados estatísticos e triagem de plágio quase instantaneamente, enquanto revisores humanos podem levar dias para realizar uma verificação comparável. Essa eficiência permite que os periódicos gerenciem taxas de submissão mais altas e reduz o tempo entre a submissão e a publicação.
2. **Objetividade e consistência aprimoradas** Os revisores humanos, apesar de sua expertise, são propensos a vieses decorrentes de crenças pessoais, familiaridade com autores ou prestígio institucional. A IA, quando treinada com base em critérios claros e padronizados, pode avaliar manuscritos sem tais influências, proporcionando uma análise consistente e imparcial. Um sistema de IA poderia avaliar a adequação do tamanho de uma amostra ou a validade de métodos estatísticos com base apenas em parâmetros predefinidos. Essa objetividade pode criar um ambiente mais equitativo, garantindo que todas as submissões sejam julgadas por seus méritos e não por fatores externos, o que é particularmente benéfico para pesquisadores em início de carreira ou aqueles de instituições menos proeminentes.
3. **Escalabilidade e suporte para revisores** A escassez de revisores por pares qualificados é um problema persistente na publicação médica, especialmente em especialidades de nicho, onde a expertise se concentra em um pequeno grupo de indivíduos. A IA pode escalar o processo de revisão auxiliando revisores menos experientes ou realizando avaliações preliminares, liberando especialistas seniores para tarefas mais complexas. Ferramentas de IA podem avaliar as submissões quanto à conformidade básica — garantindo declarações de aprovação ética, conjuntos de dados completos ou formatação adequada — permitindo que revisores humanos se concentrem no rigor científico e na inovação. Essa escalabilidade também se estende a periódicos menores ou àqueles em ambientes com recursos limitados, permitindo que mantenham altos padrões sem depender exclusivamente de voluntários humanos sobrecarregados.
4. **Detecção de erros e controle de qualidade** A IA se destaca na identificação de erros técnicos que podem escapar aos revisores humanos, especialmente em manuscritos extensos ou com grande volume de dados. Ela pode sinalizar inconsistências em figuras e tabelas, detectar dados fabricados ou manipulados (por exemplo, por meio da análise de imagens de gráficos ou fotografias) e verificar a precisão dos estudos citados. Em ensaios clínicos, por exemplo, a IA pode comparar os resultados relatados com os dados brutos enviados, detectando discrepâncias que, de outra forma, poderiam passar despercebidas. Ao aprimorar o controle de qualidade, a IA ajuda a manter a integridade da literatura médica, reduzindo o risco de pesquisas errôneas ou fraudulentas influenciarem o atendimento ao paciente.
5. **Aprendizagem contínua e adaptabilidade** Os sistemas modernos de IA, especialmente aqueles que utilizam aprendizado de máquina, podem melhorar ao longo do tempo, à medida que são expostos a mais dados e recebem feedback. Essa adaptabilidade significa que as ferramentas de IA podem se manter atualizadas com os padrões em evolução na pesquisa médica — como novas técnicas estatísticas ou diretrizes de relatórios — sem exigir um extenso retreinamento de revisores humanos.

### Desvantagens da IA na revisão por pares

1. **Compreensão contextual limitada** Embora a IA seja hábil no processamento de dados estruturados, ela enfrenta dificuldades com os aspectos diferenciados e dependentes do contexto da pesquisa médica. A revisão por pares frequentemente exige a interpretação de linguagem ambígua, a avaliação da viabilidade das implicações de um estudo ou o reconhecimento da importância de uma abordagem não convencional — tarefas que exigem intuição e experiência humanas. Por exemplo, uma IA pode julgar mal as implicações éticas de um estudo (por exemplo, a aceitabilidade de um braço placebo em um ensaio clínico) ou deixar de avaliar uma ideia inovadora que carece de precedentes em seus dados de treinamento. Essa limitação corre o risco de ignorar trabalhos inovadores ou interpretar mal cenários clínicos complexos, comprometendo a profundidade do processo de revisão.
2. **Risco de excesso de confiança e desqualificação** A conveniência da IA pode levar revisores e editores a dependerem fortemente de seus resultados, tratando-os como definitivos em vez de complementares. Se revisores humanos recorrerem à IA para tarefas como validação estatística ou verificações de metodologia, eles podem negligenciar o exame minucioso dessas áreas, potencialmente ignorando erros que a IA não consegue detectar. Com o tempo, essa dependência excessiva pode corroer as habilidades de pensamento crítico entre os revisores, um fenômeno conhecido como “desqualificação”. Desqualificação refere-se ao processo pelo qual a expertise especializada é reduzida por meio da automação, avanços tecnológicos ou mudanças nos processos de trabalho. Envolve a decomposição de tarefas complexas que antes exigiam conhecimento especializado ou treinamento em ações mais simples e repetitivas que podem ser executadas por trabalhadores ou máquinas menos qualificados. Isso pode levar a uma força de trabalho que exige menos treinamento ou experiência, ou a um declínio na habilidade artesanal. O termo é frequentemente associado a mudanças industriais e econômicas, como o surgimento de linhas de montagem ou ferramentas baseadas em IA, onde as habilidades humanas são substituídas ou desvalorizadas. Na medicina, onde falhas sutis podem ter consequências profundas, essa perda de vigilância representa um risco significativo para a qualidade da pesquisa.
3. **Viés nos dados de treinamento** Os sistemas de IA são tão bons quanto os dados com os quais são treinados, e vieses nesses dados podem perpetuar ou amplificar falhas existentes no processo de revisão. Se uma IA for treinada predominantemente em estudos de instituições ocidentais ou áreas médicas específicas, ela pode subestimar pesquisas de regiões ou especialidades sub-representadas, julgando erroneamente suas metodologias ou descartando sua relevância. Teoricamente, uma ferramenta de IA treinada em dados de países de alta renda pode sinalizar um estudo de um ambiente de poucos recursos como inadequado devido a tamanhos de amostra menores, ignorando, assim, restrições contextuais. Esse viés pode reforçar as desigualdades na publicação e distorcer a base de conhecimento médico.
4. **Erros técnicos e falsos positivos/negativos** A IA não é infalível e pode produzir erros que interrompam o processo de revisão. Falsos positivos — como acusar indevidamente um autor de plágio devido a uma formulação comum — ou falsos negativos — como ignorar uma manipulação de dados habilmente disfarçada — podem levar a resultados injustos. Por exemplo, uma IA excessivamente zelosa pode atrasar um artigo legítimo, sinalizando problemas menores, frustrando autores e obstruindo os fluxos de trabalho editoriais, enquanto uma IA com baixo desempenho pode aprovar um estudo falho, colocando em risco aplicações clínicas posteriores. Essas limitações técnicas destacam a necessidade de supervisão humana, mas também corroem a confiança na IA como ferramenta independente.
5. **Barreiras de custo e acessibilidade** O desenvolvimento, a manutenção e a implantação de sistemas sofisticados de IA exigem recursos financeiros e técnicos significativos, que podem não estar disponíveis para todos os periódicos. Custos elevados podem limitar a adoção da IA a editoras bem financiadas, deixando periódicos menores ou de acesso aberto em desvantagem. Essa disparidade pode ampliar a lacuna entre publicações de “elite” e “menores”, afetando sua capacidade de atrair submissões de qualidade ou manter padrões rigorosos. Além disso, a necessidade de atualizações contínuas para manter a IA alinhada aos avanços da ciência médica aumenta os custos, tornando-a potencialmente uma opção insustentável para algumas organizações.

Um resumo das principais vantagens e desvantagens da IA na revisão por pares é apresentado na Tabela 1 .

**Tabela 1:** – Vantagens e desvantagens da inteligência artificial na revisão por pares e na publicação acadêmica

Vantagens	Desvantagens
Eficiência e velocidade aprimoradas **:** A IA processa grandes volumes de texto rapidamente, verificando referências, analisando dados e rastreando plágio, reduzindo o tempo de revisão e atrasos na publicação.	Compreensão contextual limitada **:** A IA tem dificuldades com aspectos diferenciados da pesquisa, como interpretar linguagem ambígua ou reconhecer abordagens não convencionais, o que pode resultar na potencial perda de trabalhos inovadores.
Objetividade e consistência aprimoradas **:** A AI avalia manuscritos sem preconceitos pessoais, usando critérios padronizados para garantir um julgamento equitativo com base no mérito.	Risco de excesso de confiança e desqualificação **:** A dependência excessiva da IA pode reduzir o pensamento crítico entre os revisores, levando à perda de conhecimento especializado e vigilância.
Escalabilidade e suporte para revisores **:** A IA auxilia revisores menos experientes e lida com tarefas preliminares, permitindo que especialistas se concentrem em avaliações complexas e possibilitando que periódicos menores mantenham altos padrões.	Viés nos dados de treinamento **:** IA treinada em conjuntos de dados tendenciosos pode subestimar pesquisas de regiões ou especialidades sub-representadas, reforçando as desigualdades na publicação.
Detecção de erros e controle de qualidade **:** A IA identifica erros técnicos, inconsistências e dados manipulados, melhorando a integridade da literatura médica.	Erros técnicos e falsos positivos/negativos **:** A IA pode produzir erros como falsas acusações de plágio ou deixar passar manipulações sutis, interrompendo o processo de revisão.
Aprendizagem contínua e adaptabilidade **:** A IA melhora com o tempo, mantendo-se atualizada com os padrões de pesquisa em evolução, sem necessidade de retreinamento humano extensivo.	Barreiras de custo e acessibilidade **:** Os altos custos de desenvolvimento e manutenção de IA podem limitar a adoção a periódicos bem financiados, aumentando assim as disparidades na qualidade das publicações.

IA: inteligência artificial.

### Aspectos éticos da IA como ferramenta para revisão por pares de artigos médicos

A integração da IA no processo de revisão por pares de artigos médicos introduz uma série de considerações éticas que vão além de suas vantagens e limitações técnicas. Essas preocupações envolvem justiça, transparência, responsabilização, equidade e a preservação da supervisão humana em um campo em que os riscos — saúde e bem-estar humanos — são excepcionalmente altos.

Embora a IA ofereça benefícios inegáveis, sua integração deve ser pautada por princípios que priorizem a confiança, a justiça e o contexto humano da medicina. Os periódicos devem adotar a IA de forma transparente, como um complemento e não como um substituto para a expertise humana, e garantir que seu uso não aprofunde desigualdades ou obscureça responsabilidades. Ao abordar esses desafios éticos de frente, a comunidade médica pode aproveitar o potencial da IA salvaguardando os valores que sustentam a investigação científica e o atendimento ao paciente. Em última análise, a questão não é se a IA pode auxiliar na revisão por pares, mas como ela pode fazê-lo de forma a manter os mais altos padrões éticos.

### Justiça para com os autores e a comunidade científica

Uma das principais questões éticas é se é justo submeter o trabalho de um autor à avaliação por uma máquina em vez de um especialista humano. A revisão por pares tem sido, há muito tempo, um processo conduzido por humanos, fundamentado na expertise, empatia e raciocínio crítico de cientistas que entendem os desafios da pesquisa. Um sistema de IA, por mais sofisticado que seja, carece da experiência vivida e da intuição que os revisores humanos trazem para a mesa. Por exemplo, um pesquisador que propõe uma nova hipótese que desafia a sabedoria convencional pode ser injustamente penalizado por uma IA treinada em padrões existentes, o que poderia sinalizar o trabalho como um caso atípico ou anomalia em vez de reconhecer seu potencial brilhantismo. Isso levanta a questão de se os autores merecem um processo de revisão que valorize plenamente o contexto humano de seu trabalho, especialmente na medicina, onde a inovação pode salvar vidas.

Além disso, a justiça se estende à consistência. Se alguns periódicos adotarem revisão assistida por IA enquanto outros dependem exclusivamente de revisores humanos, podem surgir disparidades no escrutínio. Um artigo rejeitado por um processo baseado em IA pode ter sido aceito sob revisão humana, ou vice-versa, potencialmente afetando a trajetória profissional do autor, as oportunidades de financiamento ou a capacidade de influenciar a prática clínica. Essa variabilidade desafia o princípio de tratamento equitativo em toda a comunidade científica.

### Transparência e confiança

A transparência é um pilar fundamental da prática científica ética, mas o uso de IA na revisão por pares complica esse ideal. Os periódicos devem divulgar quando as ferramentas de IA são utilizadas e, em caso afirmativo, em que medida? Se um sistema de IA sinalizar um erro estatístico ou potencial plágio, os autores têm o direito de saber como essa determinação foi feita — no entanto, a natureza de “caixa preta” de muitos algoritmos de IA dificulta a explicação das decisões em termos humanos. Sem uma divulgação clara, autores e leitores podem questionar a legitimidade do processo de revisão, suspeitando que julgamentos opacos de máquinas substituíram a rigorosa avaliação humana.

Essa falta de transparência pode minar a confiança, um ativo essencial na publicação médica. Os leitores dependem de periódicos revisados por pares para fornecer pesquisas confiáveis e verificadas que embasam decisões clínicas. Se perceberem que a IA está moldando silenciosamente os resultados — talvez rejeitando estudos válidos ou aprovando estudos falhos devido a peculiaridades algorítmicas —, a confiança na literatura pode vacilar. Do ponto de vista ético, os periódicos devem encontrar um equilíbrio entre a eficiência da IA e a necessidade de manter um processo aberto e confiável, possivelmente exigindo relatórios detalhados sobre o papel e as limitações da IA.

### Responsabilidade e responsabilização

Uma questão ética particularmente espinhosa é a da responsabilização. Na revisão por pares tradicional, revisores e editores humanos são responsáveis por seus julgamentos. Se um estudo falho escapa e causa danos — digamos, ao promover um tratamento ineficaz — a comunidade científica pode rastrear o erro até indivíduos ou processos específicos e corrigi-lo. No entanto, quando a IA está envolvida, essa cadeia de responsabilização se torna confusa. Se uma ferramenta de IA não detecta uma falha crítica em um artigo médico, quem é o culpado? Os desenvolvedores que projetaram o algoritmo? O periódico que o implementou? O revisor humano que confiou em seus resultados? A IA não pode ser responsabilizada moral ou legalmente, deixando uma lacuna que pode minar a justiça e o aprendizado com os erros.

Essa questão se torna ainda mais urgente na medicina, onde erros podem ter consequências de vida ou morte. Considere um cenário em que uma revisão assistida por IA aprova um estudo com falhas metodológicas sutis, resultando em uma diretriz clínica amplamente adotada, mas potencialmente prejudicial. A difusão de responsabilidades — entre humanos e máquinas — pode atrasar ações corretivas e agravar os danos. Do ponto de vista ético, o uso de IA exige uma estrutura clara de supervisão, garantindo que os revisores humanos mantenham a autoridade máxima e que os periódicos estabeleçam protocolos para auditar as decisões de IA.

### Equidade e acesso

O princípio ético da equidade levanta questões sobre quem se beneficia da IA na revisão por pares e quem pode ficar para trás. Ferramentas avançadas de IA frequentemente exigem investimentos financeiros significativos, recursos computacionais e expertise para serem implementadas de forma eficaz. Periódicos de prestígio e bem financiados podem adotar prontamente essas tecnologias, aumentando sua eficiência e reputação, enquanto periódicos menores ou aqueles em ambientes com poucos recursos podem ter dificuldade para acompanhar o ritmo. Isso pode exacerbar as disparidades existentes na publicação acadêmica, onde pesquisadores já marginalizados — como aqueles de países em desenvolvimento ou instituições subfinanciadas — enfrentam barreiras adicionais para ter seus trabalhos revisados e publicados.

Tais desigualdades podem ter efeitos colaterais sobre o conhecimento médico. Se periódicos assistidos por IA publicarem desproporcionalmente pesquisas de regiões ou instituições com bons recursos, a comunidade médica global poderá perder perspectivas diversas, especialmente de áreas onde as doenças são endêmicas, mas a infraestrutura de pesquisa é limitada. Do ponto de vista ético, a implantação da IA não deve exacerbar essas lacunas; em vez disso, esforços devem ser feitos para garantir amplo acesso, talvez por meio do uso de ferramentas de IA de código aberto ou de subsídios para periódicos menores.

### Preservação do julgamento humano

Em sua essência, o debate ético gira em torno do papel do julgamento humano na ciência. A medicina não é meramente uma área técnica, mas profundamente humana, envolvendo dilemas éticos, experiências de pacientes e implicações sociais que a IA não consegue compreender plenamente. A revisão por pares visa tanto avaliar o peso moral de um estudo — seu potencial para curar ou causar danos — quanto verificar seus dados. Uma IA pode ser excelente em detectar erros estatísticos, mas falhar ao avaliar se as conclusões de um estudo ultrapassam suas evidências de forma a induzir médicos ou formuladores de políticas a erro.

O uso da IA corre o risco de diminuir esse elemento humano, transformando a revisão por pares em uma lista de verificação mecânica em vez de um processo reflexivo e deliberativo. Do ponto de vista ético, a comunidade científica deve questionar se a eficiência justifica deixar de lado a empatia, a criatividade e a sensibilidade ética proporcionadas por revisores humanos. Uma abordagem equilibrada — na qual a IA lida com tarefas rotineiras, mas os humanos mantêm o controle sobre os julgamentos interpretativos e morais — parece essencial para preservar a integridade da pesquisa médica.

### Riscos interligados ao enviar manuscritos para sistemas de IA

Quando um manuscrito é publicado — seja por um revisor verificando erros, um editor resumindo o conteúdo ou um periódico testando plágio — os dados geralmente saem do ambiente controlado dos servidores da editora ou instituição. Em vez disso, são transmitidos para plataformas externas, normalmente gerenciadas por empresas privadas. Essas plataformas podem não estar vinculadas aos mesmos padrões rigorosos de confidencialidade dos periódicos acadêmicos, que tradicionalmente tratam manuscritos não publicados como sagrados até a publicação formal. O próprio ato de publicar transfere o controle do autor e da editora, introduzindo uma terceira parte que pode não priorizar ou garantir o mesmo nível de privacidade.

Um perigo específico é que os dados enviados podem ser retidos para fins de treinamento. Muitos sistemas de IA melhoram seu desempenho incorporando entradas do usuário em seus conjuntos de dados de treinamento. Se um manuscrito contendo novas descobertas de pesquisa, dados experimentais ou metodologias inovadoras for inserido em um sistema desse tipo, ele poderá ser armazenado indefinidamente nos servidores do provedor. Pior ainda, poderá ser processado e integrado à base de conhecimento da IA potencialmente ressurgindo em resultados futuros.

O segundo grande risco — vulnerabilidade a vazamentos ou ataques de hackers — agrava esse problema. Um hacker que obtivesse acesso aos servidores de um provedor de IA poderia extrair manuscritos não publicados, notas de revisores ou identidades de autores, especialmente em processos com dupla ocultação, onde o anonimato é crucial. Mesmo sem intenção maliciosa, vazamentos acidentais poderiam ocorrer — por exemplo, por meio de um servidor mal configurado ou de um erro de funcionário —, liberando material confidencial para o domínio público. Violações de confidencialidade poderiam minar a confiança no sistema de revisão por pares, dissuadir autores de submeter artigos a periódicos que utilizam IA ou gerar disputas legais sobre propriedade intelectual. Para áreas como farmacêutica ou tecnologia, onde o sigilo pré-publicação é fundamental, os riscos são ainda maiores.

### Alcançando um equilíbrio

A análise de artigos médicos por pares assistida por IA oferece vantagens inegáveis, incluindo maior eficiência, objetividade e escalabilidade. No entanto, suas limitações — como falta de compreensão contextual, potencial para excesso de confiança e vieses técnicos — destacam a necessidade de uma implementação cuidadosa. Do ponto de vista ético, o uso de IA é permissível e até benéfico, desde que seja transparente, complementar à expertise humana e projetado para minimizar desigualdades e erros.

Em vez de substituir revisores humanos, a IA deve servir como uma ferramenta colaborativa, ampliando suas capacidades e preservando o julgamento humano essencial à ciência médica. Os periódicos devem estabelecer diretrizes claras para o papel da IA garantindo a responsabilização e mantendo a confiança da comunidade científica. Com essas salvaguardas em vigor, a IA pode aprimorar o processo de revisão por pares sem comprometer sua integridade, promovendo, em última análise, a qualidade e a confiabilidade da pesquisa médica em um mundo cada vez mais complexo.
