# Supplementary material for: Assistência de Inteligência Artificial na Análise de Artigos Médicos por Pares: Vantagens, Desvantagens e Considerações Éticas
Source: Arq Bras Cardiol. 2025 Aug 12;122(8):e20250315. [Article in Portuguese] doi: 10.36660/abc.20250315 (PMC12677890; doi:10.36660/abc.20250315)
Supplement: Translation

# Translation: Artificial Intelligence Assistance in the Peer Review Analysis of Medical Articles: Advantages, Disadvantages, and Ethical Considerations

**DOI:** 10.36660/abc.20250315i

**Authors:** André Zimerman, Andre d’Avila

**Affiliations:** 1 Brazil MOVE Academic Research Organization, Hospital Moinhos de Vento, Porto Alegre, RS - Brazil; 2 Brazil Hospital SOS Cardio, Florianópolis, SC – Brazil

**Keywords:** Peer Review, Artificial Intelligence, Ethics, Publications

Recently, the authors of this “Viewpoint” article were invited to peer review a manuscript for a prestigious international cardiovascular journal. After dedicating a few days to the analysis, they decided, out of curiosity, to upload the manuscript into a commercially available large language model with artificial intelligence (AI) capabilities, using the following prompt: “Analyze and Criticize the Attached File.” Both reviewers were astonished by the response generated within seconds by the AI algorithm — its depth and precision exceeded their expectations, with some insights proving complementary to their own. As a result, they incorporated these elements into the official review submitted to the journal.

This experience, however, raised another question: should they acknowledge the assistance provided by the AI algorithm? Uncertain of the answer, they consulted other physicians who regularly review manuscripts for high-impact journals, and their opinions varied widely. Some argued that AI is merely a tool to enhance efficiency, requiring no acknowledgment—reviewers are accountable for the final content and should use whatever strategies enable them to achieve the best outcome. Others insisted that failing to disclose AI assistance would be unethical.

To resolve the dilemma, the authors yet again turned to AI itself for insight into its role in the peer review process of medical manuscripts. The response, presented here, required minimal editorial adjustments from the authors. *Whenever deemed necessary, the authors’ inputs are reported throughout the manuscript in italics.*

## AI talks about the role of AI as a reviewer of medical papers:

The peer review process is a cornerstone of scientific integrity, particularly in the field of medicine, where rigorous evaluation ensures that published research is credible, reproducible, and safe for clinical application. However, the growing volume of medical articles submitted for publication, coupled with the complexity of modern research, has strained the traditional peer review system.

The use of AI in the peer review of medical articles represents a transformative opportunity to address the mounting challenges of academic publishing in a field where accuracy, timeliness, and reliability are paramount. However, alongside its potential benefits, AI introduces significant drawbacks and notable challenges. This essay examines the benefits and shortcomings of AI in the peer review analysis of medical articles, as well as the ethical implications of its integration.

### Advantages of AI in peer review

1. **Enhanced efficiency and speed** The sheer volume of medical research submitted for publication has risen steeply, often overwhelming human reviewers and delaying the dissemination of critical findings. AI can process vast amounts of text rapidly, performing tasks such as checking references, analyzing statistical data, and screening for plagiarism almost instantaneously, while human reviewers might take days to perform a comparable check. This efficiency allows journals to handle higher submission rates and reduces the time from submission to publication.
2. **Improved objectivity and consistency** Human reviewers, despite their expertise, are prone to biases stemming from personal beliefs, familiarity with authors, or institutional prestige. AI, when trained on clear and standardized criteria, can evaluate manuscripts without such influences, providing a consistent and impartial analysis. An AI system could assess the adequacy of a sample size or the validity of statistical methods based solely on predefined benchmarks. This objectivity can create a more equitable environment, ensuring that all submissions are judged on their merits rather than extraneous factors, which is particularly beneficial for early-career researchers or those from less prominent institutions.
3. **Scalability and support for reviewers** The shortage of qualified peer reviewers is a persistent issue in medical publishing, especially in niche specialties where expertise is concentrated among a small pool of individuals. AI can scale the review process by assisting less experienced reviewers or handling preliminary evaluations, freeing up senior experts for more complex tasks. AI tools might screen submissions for basic compliance—ensuring ethical approval statements, complete datasets, or proper formatting—allowing human reviewers to focus on scientific rigor and innovation. This scalability also extends to smaller journals or those in resource-limited settings, enabling them to maintain high standards without relying solely on overburdened human volunteers.
4. **Error detection and quality control** AI excels at identifying technical errors that might elude human reviewers, particularly in large or data-heavy manuscripts. It can flag inconsistencies in figures and tables, detect fabricated or manipulated data (e.g., through image analysis of graphs or photographs), and verify the accuracy of cited studies. In clinical trials, for instance, AI could cross-check reported outcomes against raw data submissions, catching discrepancies that might otherwise go unnoticed. By enhancing quality control, AI helps uphold the integrity of the medical literature, reducing the risk of erroneous or fraudulent research influencing patient care.
5. **Continuous learning and adaptability** Modern AI systems, particularly those that leverage machine learning, can improve over time as they are exposed to more data and receive feedback. This adaptability means that AI tools can stay current with evolving standards in medical research—such as new statistical techniques or reporting guidelines—without requiring extensive retraining of human reviewers.

### Disadvantages of AI in peer review

1. **Limited contextual understanding** While AI is adept at processing structured data, it struggles with the nuanced, context-dependent aspects of medical research. Peer review often requires interpreting ambiguous language, assessing the feasibility of a study’s implications, or recognizing the significance of an unconventional approach—tasks that demand human intuition and experience. For example, an AI might misjudge a study’s ethical implications (e.g., the acceptability of a placebo arm in a trial) or fail to appreciate a paradigm-shifting idea that lacks precedent in its training data. This limitation risks overlooking groundbreaking work or misinterpreting complex clinical scenarios, undermining the depth of the review process.
2. **Risk of over-reliance and deskilling** The convenience of AI could lead reviewers and editors to depend heavily on its outputs, treating them as definitive rather than supplementary. If human reviewers defer to AI for tasks such as statistical validation or methodology checks, they may neglect to scrutinize these areas themselves, potentially missing errors that the AI fails to detect. Over time, this over-reliance could erode critical thinking skills among reviewers, a phenomenon known as “deskilling.” Deskilling refers to the process by which skilled expertise is reduced through automation, technological advancements, or changes in work processes. It involves breaking down complex tasks that once required specialized knowledge or training into simpler, repetitive actions that less-skilled workers or machines can perform. This can lead to a workforce requiring less training or experience, or a decline in craftsmanship. The term is often associated with industrial and economic shifts, such as the rise of assembly lines or AI-driven tools, where human skills are replaced or devalued. In medicine, where subtle flaws can have profound consequences, this loss of vigilance poses a significant risk to the quality of research.
3. **Bias in training data** AI systems are only as good as the data they are trained on, and biases in that data can perpetuate or amplify existing flaws in the review process. If an AI is trained predominantly on studies from Western institutions or specific medical fields, it might undervalue research from underrepresented regions or specialties, misjudging their methodologies or dismissing their relevance. Theoretically, an AI tool trained on high-income country data might flag a study from a low-resource setting as inadequate due to smaller sample sizes, thereby ignoring contextual constraints. This bias could reinforce inequities in publishing and skew the medical knowledge base.
4. **Technical errors and false positives/negatives** AI is not infallible and can produce errors that disrupt the review process. False positives—such as wrongly accusing an author of plagiarism due to common phrasing—or false negatives—such as missing a cleverly disguised data manipulation—can lead to unjust outcomes. For example, an overzealous AI might delay a legitimate paper by flagging minor issues, frustrating authors, and clogging editorial workflows, while an underperforming AI might approve a flawed study, endangering downstream clinical applications. These technical limitations highlight the need for human oversight, but they also erode confidence in AI as a standalone tool.
5. **Cost and accessibility barriers** Developing, maintaining, and deploying sophisticated AI systems requires significant financial and technical resources, which may not be available to all journals. High costs could limit AI adoption to well-funded publishers, leaving smaller or open-access journals at a disadvantage. This disparity might widen the gap between “elite” and “lesser” publications, affecting their ability to attract quality submissions or maintain rigorous standards. Additionally, the need for ongoing updates to keep AI aligned with advancing medical science adds to the expense, potentially making it an unsustainable option for some organizations.

A summary of the main advantages and disadvantages of AI in peer review is presented in Table 1 .

**Table 1:** – Advantages and Disadvantages of Artificial Intelligence in Peer Review and Academic Publishing

Advantages	Disadvantages
Enhanced efficiency and speed: AI processes large volumes of text quickly, checking references, analyzing data, and screening for plagiarism, reducing review time and publication delays.	Limited contextual understanding: AI struggles with nuanced aspects of research, such as interpreting ambiguous language or recognizing unconventional approaches, which can result in the potential missing of groundbreaking work.
Improved objectivity and consistency: AI evaluates manuscripts without personal biases, using standardized criteria to ensure equitable judgment based on merit.	Risk of over-reliance and deskilling: Over-dependence on AI may reduce critical thinking among reviewers, leading to a loss of expertise and vigilance.
Scalability and support for reviewers: AI assists less experienced reviewers and handles preliminary tasks, allowing experts to focus on complex evaluations and enabling smaller journals to maintain high standards.	Bias in training data: AI trained on biased datasets may undervalue research from underrepresented regions or specialties, reinforcing publishing inequities.
Error detection and quality control: AI identifies technical errors, inconsistencies, and manipulated data, enhancing the integrity of medical literature.	Technical errors and false positives/negatives: AI can produce errors like false plagiarism accusations or miss subtle manipulations, disrupting the review process.
Continuous learning and adaptability: AI improves over time, staying current with evolving research standards without extensive human retraining.	Cost and accessibility barriers: The high costs of AI development and maintenance may limit adoption to well-funded journals, thereby widening disparities in publishing quality.

AI: artificial intelligence.

### Ethical aspects of AI as a tool for peer review of medical articles

The integration of AI into the peer review process of medical articles introduces a range of ethical considerations that extend beyond its technical advantages and limitations. These concerns touch on fairness, transparency, accountability, equity, and the preservation of human oversight in a field where the stakes—human health and well-being—are exceptionally high.

While AI offers undeniable benefits, its integration must be guided by principles that prioritize trust, justice, and the human context of medicine. Journals should adopt AI transparently, as a supplement rather than a substitute for human expertise, and ensure that its use does not deepen inequities or obscure responsibility. By addressing these ethical challenges head-on, the medical community can harness AI’s potential while safeguarding the values that underpin scientific inquiry and patient care. Ultimately, the question is not whether AI can assist in peer review but how it can do so in a way that upholds the highest ethical standards.

### Fairness to authors and the scientific community

One of the primary ethical questions is whether it is fair to subject an author’s work to evaluation by a machine rather than a human expert. Peer review has long been a human-driven process grounded in the expertise, empathy, and critical reasoning of scientists who understand the challenges of research. An AI system, no matter how sophisticated, lacks the lived experience and intuition that human reviewers bring to the table. For instance, a researcher proposing a novel hypothesis that challenges conventional wisdom might be unfairly penalized by an AI trained on existing patterns, which could flag the work as an outlier or anomaly rather than recognizing its potential brilliance. This raises the issue of whether authors deserve a review process that fully appreciates the human context of their work, especially in medicine, where innovation can save lives.

Moreover, fairness extends to consistency. If some journals adopt AI-assisted review while others rely solely on human reviewers, disparities in scrutiny could emerge. A paper rejected by an AI-driven process might have been accepted under human review, or vice versa, potentially affecting an author’s career trajectory, funding opportunities, or ability to influence clinical practice. This variability challenges the principle of equitable treatment across the scientific community.

### Transparency and trust

Transparency is a cornerstone of ethical scientific practice, yet the use of AI in peer review complicates this ideal. Should journals disclose when AI tools are used, and if so, to what extent? If an AI system flags a statistical error or potential plagiarism, authors have a right to know how that determination was made—yet the “black box” nature of many AI algorithms makes it difficult to explain decisions in human terms. Without clear disclosure, authors and readers might question the legitimacy of the review process, suspecting that opaque machine judgments have replaced rigorous human evaluation.

This lack of transparency could erode trust, a critical currency in medical publishing. Readers rely on peer-reviewed journals to provide credible, vetted research that informs clinical decisions. If they perceive that AI is silently shaping outcomes—perhaps rejecting valid studies or approving flawed ones due to algorithmic quirks—confidence in the literature could falter. Ethically, journals must strike a balance between the efficiency of AI and the imperative to maintain an open and trustworthy process, possibly by mandating detailed reporting on AI’s role and limitations.

### Accountability and responsibility

A particularly thorny ethical issue is accountability. In traditional peer review, human reviewers and editors bear responsibility for their judgments. If a flawed study slips through and causes harm—say, by promoting an ineffective treatment—the scientific community can trace the error back to specific individuals or processes and address it. However, when AI is involved, this chain of accountability becomes blurred. If an AI tool misses a critical flaw in a medical article, who is at fault? The developers who designed the algorithm? The journal that deployed it? The human reviewer who trusted its output? AI cannot be held morally or legally accountable, leaving a gap that could undermine justice and learning from mistakes.

This issue becomes even more pressing in medicine, where errors can have life-or-death consequences. Consider a scenario in which an AI-assisted review approves a study with subtle methodological flaws, resulting in a widely adopted but potentially harmful clinical guideline. The diffusion of responsibility—between humans and machines—could delay corrective action and exacerbate harm. Ethically, the use of AI demands a clear framework for oversight, ensuring that human reviewers retain ultimate authority and that journals establish protocols for auditing AI decisions.

### Equity and access

The ethical principle of equity raises questions about who benefits from AI in peer review and who might be left behind. Advanced AI tools often require significant financial investment, computational resources, and expertise to implement effectively. Prestigious, well-funded journals may readily adopt these technologies, enhancing their efficiency and reputation, while smaller journals or those in low-resource settings might struggle to keep pace. This could exacerbate existing disparities in academic publishing, where already marginalized researchers—such as those from developing countries or underfunded institutions—face additional barriers to having their work reviewed and published.

Such inequities could have downstream effects on medical knowledge. If AI-assisted journals disproportionately publish research from well-resourced regions or institutions, the global medical community might miss out on diverse perspectives, particularly from areas where diseases are endemic, but research infrastructure is limited. Ethically, the deployment of AI should not exacerbate these gaps; instead, efforts should be made to ensure broad access, perhaps through the use of open-source AI tools or subsidies for smaller journals.

### Preservation of human judgment

At its core, the ethical debate hinges on the role of human judgment in science. Medicine is not merely a technical field but a deeply human one involving ethical dilemmas, patient experiences, and societal implications that AI cannot fully comprehend. Peer review is as much about assessing the moral weight of a study—its potential to heal or harm—as it is about verifying its data. An AI might excel at spotting statistical errors but falter when evaluating whether a study’s conclusions overstep its evidence in ways that could mislead clinicians or policymakers.

Using AI risks diminishing this human element, turning peer review into a mechanical checklist rather than a reflective, deliberative process. Ethically, the scientific community must ask whether efficiency justifies sidelining the empathy, creativity, and ethical sensitivity that human reviewers provide. A balanced approach—where AI handles rote tasks, but humans retain control over interpretive and moral judgments—seems essential to preserving the integrity of medical research.

### Intertwined risks of uploading manuscripts to AI systems

When a manuscript is uploaded—whether by a reviewer checking for errors, an editor summarizing content, or a journal testing for plagiarism—the data often leaves the controlled environment of the publisher’s or institution’s servers. Instead, it is transmitted to external platforms, typically managed by private companies. These platforms may not be bound by the same strict confidentiality standards as academic journals, which traditionally treat unpublished manuscripts as sacrosanct until formal publication. The act of uploading itself shifts control away from the author and publisher, introducing a third party that may not prioritize or guarantee the same level of privacy.

One specific danger is that the uploaded data might be retained for training purposes. Many AI systems improve their performance by incorporating user inputs into their training datasets. If a manuscript containing novel research findings, experimental data, or innovative methodologies is fed into such a system, it could be stored indefinitely on the provider’s servers. Worse, it might be processed and integrated into the AI’s knowledge base, potentially resurfacing in future outputs.

The second major risk—vulnerability to leaks or hacking—compounds this issue. A hacker gaining access to an AI provider’s servers could extract unpublished manuscripts, reviewer notes, or author identities, especially in double-masked processes where anonymity is critical. Even without malicious intent, accidental leaks could occur—say, through a misconfigured server or an employee error—releasing confidential material into the public domain. Confidentiality breaches could erode trust in the peer review system, deter authors from submitting to journals using AI, or spark legal disputes over intellectual property. For fields like pharmaceuticals or technology, where pre-publication secrecy is paramount, the stakes are even higher.

### Striking a balance

AI-assisted peer review analysis of medical articles offers undeniable advantages, including increased efficiency, objectivity, and scalability. However, its limitations—such as lack of contextual understanding, potential for over-reliance, and technical biases—highlight the need for careful implementation. Ethically, the use of AI is permissible and even beneficial, provided it is transparent, supplementary to human expertise, and designed to minimize inequities and errors.

Rather than replacing human reviewers, AI should serve as a collaborative tool, augmenting their capabilities while preserving the human judgment that is essential to medical science. Journals must establish clear guidelines for AI’s role, ensuring accountability and maintaining the trust of the scientific community. With these safeguards in place, AI can enhance the peer review process without compromising its integrity, ultimately advancing the quality and reliability of medical research in an increasingly complex world.
